# Recent advances in synthetic approaches for medicinal chemistry of C-nucleosides

**DOI:** 10.3762/bjoc.14.65

**Published:** 2018-04-05

**Authors:** Kartik Temburnikar, Katherine L Seley-Radtke

**Affiliations:** 1 Department of Pharmacology and Molecular Sciences, Johns Hopkins University School of Medicine, 725 N. Wolfe St. Baltimore, MD 21205, United States; 2 Department of Chemistry and Biochemistry, University of Maryland Baltimore County, 1000 Hilltop Circle, Baltimore, MD 21250, United States

**Keywords:** C-nucleosides, convergent synthesis, modular synthesis

## Abstract

C-nucleosides have intrigued biologists and medicinal chemists since their discovery in 1950's. In that regard, C-nucleosides and their synthetic analogues have resulted in promising leads in drug design. Concurrently, advances in chemical syntheses have contributed to structural diversity and drug discovery efforts. Convergent and modular approaches to synthesis have garnered much attention in this regard. Among them nucleophilic substitution at C1' has seen wide applications providing flexibility in synthesis, good yields, the ability to maneuver stereochemistry as well as to incorporate structural modifications. In this review, we describe recent reports on the modular synthesis of C-nucleosides with a focus on D-ribonolactone and sugar modifications that have resulted in potent lead molecules.

## Introduction

**Figure 1:**
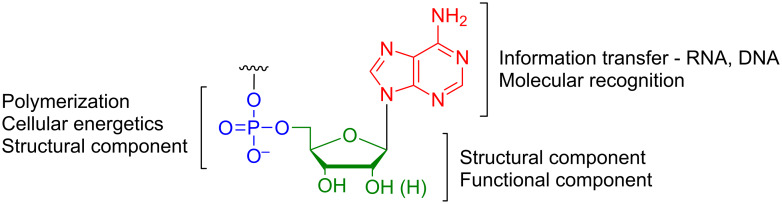
Structural components of nucleic acids. Shown is the monomeric building block of nucleic acids. Changes to the nucleotide structure can affect molecular recognition, as well as structure and function.

Nucleic acids form the genetic blueprint for all living organisms and are involved with a wide range of cellular functions [1–9]. Modifications to their chemical structure can have profound effects on structure and function of enzymes, cells and supramolecular complexes [10–22]. Nucleic acids are composed of a monomeric nucleoside unit that features an aromatic nitrogenous moiety (a nucleobase) connected to a pentose sugar, which in turn is attached to a phosphate group (Figure 1) [7]. The pentose sugar and the nucleobase are connected by a carbon–nitrogen bond that is adjacent to the sugar oxygen resulting in an hemiaminal ether bond, also known as the glycosidic bond.

Because of their key role in many biological processes, modifications to the nucleoside structure have been widely employed in the design of drugs, most notably in the fields of virology and cancer research [13–15]. Variations in the nucleoside scaffold are typically accomplished by the insertion, deletion or transposition of functional groups or atoms [23–29]. The varied properties of such modified nucleosides arise from changes in hydrogen bonding motifs, electronic effects, hydrophobic interactions, acid-base properties and chemical reactivity [25–37]. One such modification is the change in the nature of the glycosidic bond [29,37].

**Figure 2:**
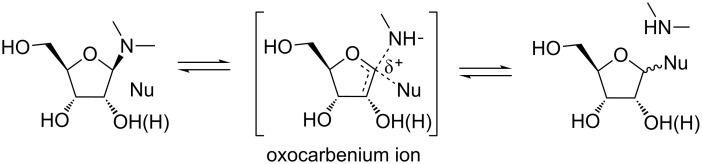
Formation of oxocarbenium ion during glycosidic bond cleavage in nucleosides [31]. The extent of leaving group stabilization and approach of the nucleophile determine charge accumulation on the sugar ring. A concerted process leads to a transition state-like species shown in the figure, while a greater accumulation of positive charge leads to an oxocarbenium ion intermediate.

Although the glycosidic bond is stable under physiological conditions, cleavage of the bond is common and is highly dependent on the nature of the nucleobase and local pH. In addition, the rate of glycosidic bond cleavage is higher for purines than pyrimdines [38–44]. Moreover, the glycosidic bond in 2'-deoxy ribonucleosides has a higher susceptibility to cleavage than in the corresponding ribonucleosides [38–41,43]. The rate of glycosidic (C–N) bond cleavage is enhanced by decreasing pH and enzymes, which modify the localized acid–base environment [31,35–36]. The C–N bond cleavage proceeds either by activation of a nucleophile that attacks C1' or by stabilization of the leaving group, which could either be the nucleobase or an oxocarbenium ion [31,36]. As such, the oxocarbenium ion is a species formed during the glycosidic bond cleavage, which may be present as an intermediate or a transition state depending upon the accumulation of the positive charge on the sugar ring (Figure 2). As a result, any change in the nucleobase–sugar connectivity (C–N) affects the formation of the oxocarbenium ion and thus influences the stability (or instability) of the nucleoside analogues.

**Figure 3:**
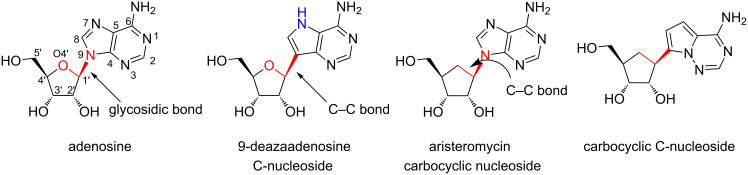
Structural modifications to nucleobase-sugar connectivity. The O–C–N bond between nucleobase and sugar defines the glycosidic bond. Replacement of nucleobase nitrogen by carbon results in C-nucleosides. A carbon (CH_2_) replacing the sugar oxygen results in carbocyclic nucleosides. When both the heteroatoms in the glycosidic bond are replaced by carbon, the resultant compounds are called carbocyclic C-nucleosides.

**Figure 4:**
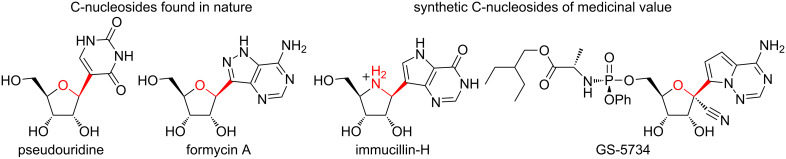
Examples of natural and synthetic C-nucleosides. Pseudouridine and formcycin are among several naturally occurring C-nucleosides that are being studied for their role in RNA biology and antibiotic properties respectively. In the recent past, synthetic C-nucleosides, such as immucillin-H and GS-5734, have shown potent activity against purine nucleoside phosphorylases (PNP) and broad spectrum antiviral activities.

Replacing the hemiaminal (O–C–N) connectivity of the canonical nucleosides with an O–C–C bond (Figure 3) results in a class of compounds called “C-nucleosides” [45–51]. Further modification to a C–C–C connectivity results in “carbocyclic C-nucleosides” (Figure 3) [52–53]. C-nucleosides feature (hetero)aryl aromatic groups such as 9-deazapurines, pyrimidines, pyridines and phenyl groups connected by a C–C bond to a sugar (or sugar mimic) as shown in Figure 4 [30,45–47,50,54–57]. The change in the nature of the glycosidic bond is accompanied by i) increased hydrolytic stability, ii) altered hydrogen bonding motifs, and iii) altered molecular recognition properties [25,29,37,58]. Because of these changes, C-nucleosides have been useful in the study of RNA and DNA processing enzymes, as well as drug design efforts and novel supramolecular structures [12,29,59].

Pseudouridine is a naturally occurring C-nucleoside that was first discovered in the 1950s [45–47,50]. Subsequently, many more C-nucleosides were discovered and their medicinal properties evaluated (Figure 4) [18,29,37,45–50,60–62]. Due to advances in synthetic methodologies over the years, the repertoire of C-nucleosides has since expanded and has enabled the discovery of clinically useful molecules. Some of the more prominent biologically active analogues that have advanced to clinical evaluations include the immucillins developed by Schramm et al, and Gilead’s antiviral pyrrolo[2,1-*f*]triazine C-nucleosides (GS-5734 and GS-6620) [32,63–65]. Thus, this review attempts to capture the progress in the synthesis efforts and subsequent drug discovery of the C-nucleosides over the past few years. In the first section, the structural and stereochemical underpinnings of nucleophilic substitutions to D-ribonolactone are discussed, a method that has seen wide applications. Next, we describe reports of different applications and structural variants that have expanded the diversity of the C-nucleosides. Finally, we discuss a modular synthetic approach to carbocyclic C-nucleosides that is also based on the nucleophilic substitution of ribonolactone.

## Review

### Nucleophilic addition to D-ribonolactone and its stereochemistry

**Figure 5:**
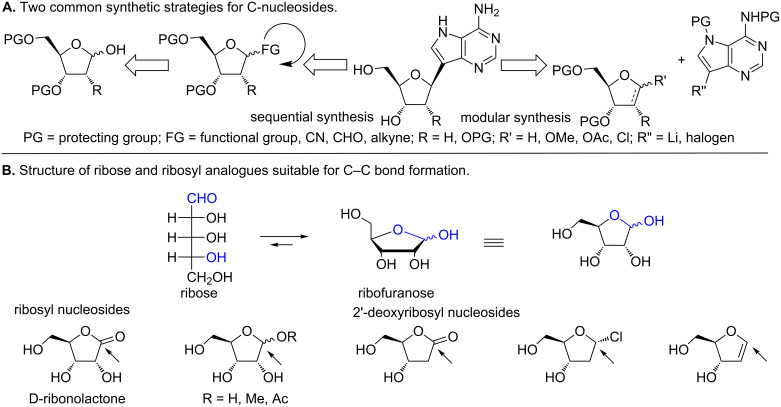
Synthetic approaches to C-nucleosides. A. Two common strategies for C-nucleoside synthesis involve functionalization at C1' and coupling of preformed sugar and heterocylic compounds. B. Structure of ribose and C1' functional groups that enable coupling reactions and synthesis of C-nucleosides.

Two prominent methods of C-nucleoside syntheses involve either i) the linear construction of a (hetero)aryl moiety on a C1'-functionalized ribose or ii) coupling of a pre-synthesized (hetero)aryl with a ribosyl moiety (Figure 5A) [48–49,62]. The C–C bond formation usually involves a functional group at C1' of the ribosyl moiety that is amenable to additional functionalization (Figure 5B). Like other nucleoside coupling approaches (other than the well-known Vorbrüggen coupling reaction [66], the synthesis of C-nucleosides typically gives a mixture of stereoisomers (α and β) at the anomeric carbon [48–49,54,62,67–68]. Since the naturally occurring nucleosides (and most biologically active nucleosides) are β-anomers, achieving 100% stereospecificity in C–C bond formation is an important goal, but often difficult to attain [62].

**Figure 6:**
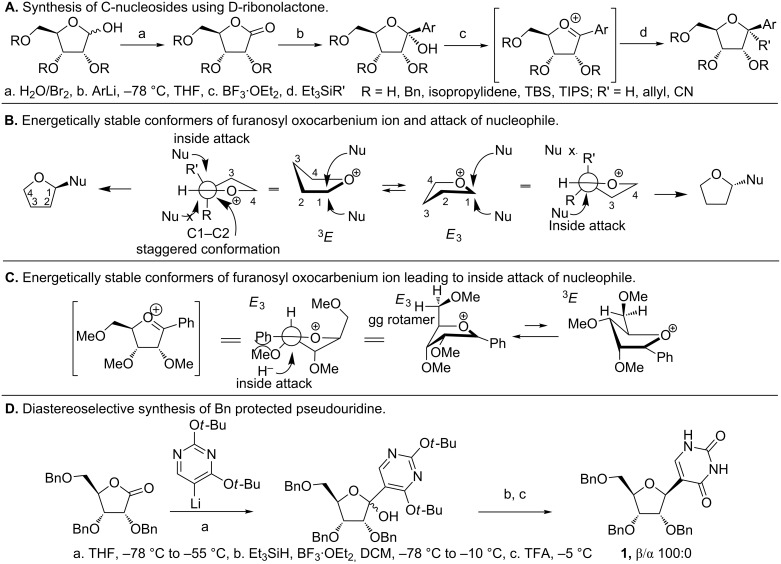
Steroselective C-nucleoside synthesis using D-ribonolactone. A. Nucleophilic substitution of D-ribonolactone results in an oxocarbenium ion intermediate. B and C. Functional groups at C2', C3' and C5' stabilize the charged sugar ring and direct the approach of nucleophile to affect the stereochemical outcome of oxocarbenium ion reduction. D. Stereoselective synthesis of protected pseudouridine [80–81].

Among the aforementioned approaches for C-nucleoside syntheses, the coupling of (hetero)aryls to the ribosyl moiety has seen the widest application [52–53,58,62–65,69–77]. This can be ascribed to the modular nature of syntheses that allows for simultaneous alterations in the sugar and the nucleobase to generate diverse analogues in a facile manner. Within this approach, nucleophilic substitution of ribonolactone (Figure 6A) has garnered much attention [61–63,69–75,78]. Ribonolactone typically with its hydroxy groups protected, is amenable to nucleophilic substitutions [78–79]. Use of C-nucleophiles such as lithiated (hetero)aryls leads to C-C bond via a lactol intermediate (Figure 6A). Subsequent deoxygenation of the C1'–OH by Lewis acids (e.g., BF_3_·OEt_2_) results in an oxocarbenium ion [62,70,80–83]. Reduction of this intermediate by various silanes gives C-nucleosides resembling the canonical nucleosides [82–83]. The stereochemical fate of oxocarbenium ion reduction is dictated by the conformation and stability of the oxocarbenium ion, which in turn, is affected by the nature of the C2', C3' and C5' substituents [80–81].

Codée and coworkers elaborated on the mechanism and stereochemistry of this reaction by calculating the energies of different oxocarbenium conformers using a free energy surface (FES) mapping method [80–81]. These studies were based on the Woerpel’s model comprising of two stable conformers, namely ^3^*E* and *E*_3_, in equilibrium (Figure 6B) [84–85]. The nucleophile approaches from the side presenting the least number of eclipsing interactions with the C2' substituent (Figure 6B) [80]. Examining the energies of the various conformers of the permethylated furanosyl oxocarbenium intermediate revealed that the *E*_3_ conformer with the C5'–OMe oriented over the positively charged furanosyl ring (Figure 6C) has a large stabilizing effect due to C5'–O5 dipole interactions. In addition, the C2' pseudoequatorial methoxy and C3' pseudoaxial methoxy groups further stabilize the intermediate in *E*_3_ conformer, thereby favoring the *E*_3_ confomer over the ^3^*E*. In the case of an anomeric phenyl group (Ph, Figure 6C), stabilization of the positive charge (C=O^+^) through conjugation, via parallel alignment, helps to overcome the unfavorable steric interactions between the C2'–OMe and the Ph group [81]. Because *E*_3_ is the favored conformer, an inside attack of the nucleophile (H^−^) results in an α orientation in the final product, which is evident from the the synthesis of **1 (**OBn-substituted Pseudouridine, Figure 6D). Despite the greater stability of the *E*_3_ conformer, it is the faster reacting conformer (*E*_3_ or ^3^*E*) that ultimately affects the ratio of diastereomers in the final product [80]. This difference in reactivity results in the differences in various α/β mixtures obtained during the synthesis of C-nucleosides using the D-ribonolactone approach.

### Antiviral C-nucleosides

**Figure 7:**
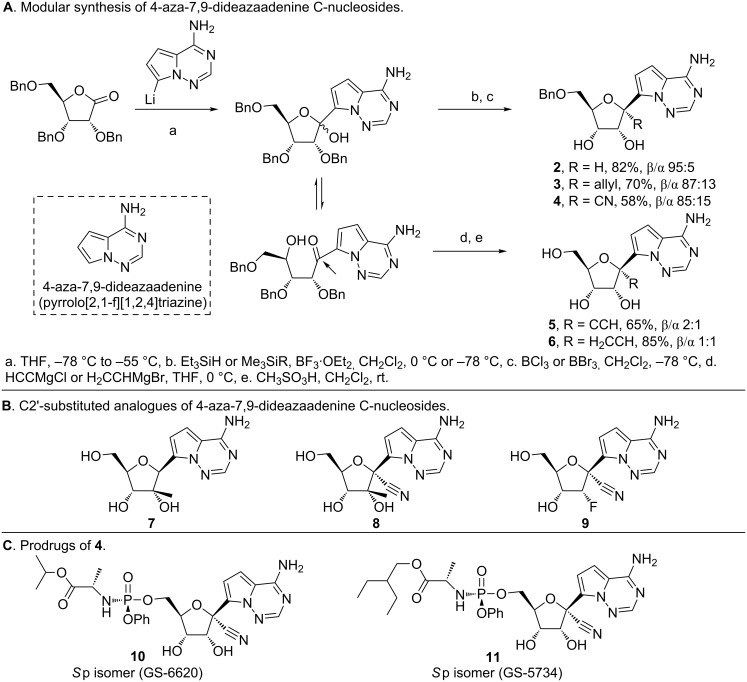
Synthesis of C1'-substituted 4-aza-7,9-dideazaadenine C-nucleosides [63–65,69–70]. A. Reaction of D-ribonolactone and lithiated heterocycle leading to C1'-substituted C-nucleosides. Steroechemical scrambling is observed when ring opened form of ribose is employed during nucleophilic substitution. B. Synthesis of C2' C-nucleosides using analogous D-ribonolactones. C. Masked C-nucleoside monophosphates exhibiting potent antiviral activity and clinical utility.

The formation of the lactol and oxocarbenium ion illustrated in Figure 6 also presents the possibility of C1' di-substitution, which was exploited by researchers at Gilead in the discovery of the potent antiviral 4-aza-7,9-dideazaadenine (pyrrolo[2,1-*f*][1,2,4]triazine) C-nucleosides (Figure 7) [63–65,69–70,77]. The synthesis of 4-aza-7,9-dideazaadenine C-nucleoside **2** (Figure 7A) was first reported by Patil et al. [86] using a sequential approach. In contrast, scientists at Gilead treated perbenzylated ribonolactone with lithiated 4-aza-7,9-dideazaadenine to obtain the lactol intermediate (Figure 7A) [63,65,69–70]. Deoxygenation of the lactol intermediate by BF_3_·OEt_2_ resulted in the oxocarbenium ion, which was then reduced using triethylsilane to obtain **2**. Replacing triethylsilane with allyl trimethylsilane and trimethylsilyl cyanide gave C1'-allyl (**3**) and C1'-cyano (**4**) substitutions respectively. A β/α ratio of 95:5, 87:13 and 89:11 was observed for **2, 3** and **4**, respectively, which was sensitive to the reaction temperature and the reagents used [69–70]. A marked difference in diastereomeric purity was observed when the open form of the ribofuranose ring (which exists in equilibrium with the ring closed form), was exploited for C1' substitution using Grignard reagents [69]. Acid-catalyzed dehydration resulted in a diastereomeric mixture of C1'-disubstituted products **5** and **6** with an observed β/α ratio of 2:1 and 1:1, respectively. Similarily, C2'-substituted ribonolactones were employed in the synthesis of 2'-β-Me analogues **7** and **8** and the 2'-deoxy-2'-fluoro **9** (Figure 7B) [65].

The 1'-α-H analogue **2** was reported to exhibit inhibitory activity against neoplastic cell lines [86]. This scaffold was later elaborated by Gilead to discover broad spectrum activity of the related C-nucleosides (**3**–**11**) against viruses from the *Flaviviridae*, *Orthomyxoviridae*, *Paramyxoviridae* and *Coronaviridae* families [63–65,69–70]. Cell-based assays revealed potent activity for compounds **2**–**9** against various viruses including Ebola (EBOV, *Filoviridae*), respiratory syncytial virus (RSV, *Pneumoviridae*) and the hepatitis-C virus (HCV, *Flaviviridae*) family [63,65,69]. Through structure activity relationship studies, the 1'-CN compound **4** emerged as a compound with activity against EBOV, HCV and RSV [65,69]. It is active against EBOV in human microvascular endothelial cells and RSV with low cytotoxicity towards Huh-7, HEp-2 and MT4 cells. Moreover, the triphosphate of **4** selectively inhibits, HCV RdRp and RSV RdRp over human RNA Pol II and DNA polymerases (α, β, γ) [65]. The 2'-Me compound **7** as its triphosphate (TP) shows anti-HCV activity in replicon assays [77]. However, **7**-TP serves as a substrate for mitochondrial RNA polymerase, thereby causing toxicity in rats [63]. The 2'-F and 2'-β-Me compounds **8** and **9** are active against the HCV, but lack activity against EBOV and RSV in cell-based assays [65]. The pharmacokinetic properties of **4** were improved by converting it to the masked monophosphates (**10** and **11**, Figure 7C), which serves to facilitate transport into the infected cells, and conversion to the active triphosphate form, thereby leading to high and persistent levels [63–65]. The 2-ethylbutyl L-alanine phosphoramidate prodrug (Sp isomer, GS-5734, **11**) increases the loading of macrophages derived from human monocytes over its unmasked analogue [64]. It was also observed that intravenous administration of the prodrug leads to increased liver loading (as the triphosphate) in hamsters compared to oral dosing [63].

**Figure 8:**
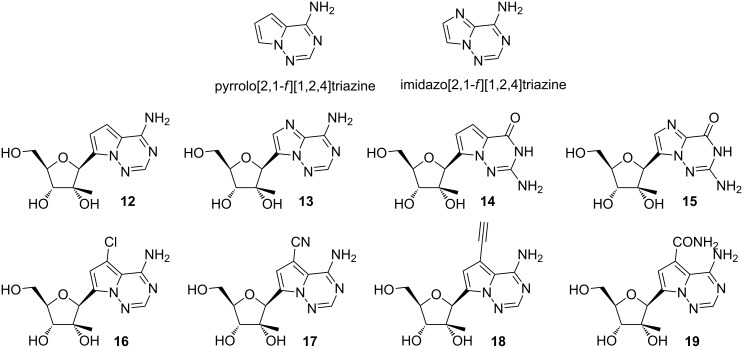
Pyrrolo- and imidazo[2,1-*f*][1,2,4]triazine C-nucleosides. A series of sugar- and nucleobase-substituted C-nucleosides were synthesized via nucleophilic substitution of D-ribonolactone for structure–activity relationship [71–72].

Draffan et al. synthesized a series of 2'-β-Me analogues of pyrrolo- and imidazo[2,1-*f*][1,2,4]triazine C-nucleosides using a 2'-β-Me lactone that mimic adenosine and guanosine (**12**–**19**, Figure 8) [71–72]. The adenine analogues of pyrrolo- and imidazo[2,1-*f*][1,2,4]triazine were active as nucleosides in HCV1b RNA replication assays, and as triphosphates they inhibit the NS5B polymerase as did the triphosphates of the guanosine analogues [71]. The library of adenosine analogues was further expanded by introducing functional groups at C7 (**16**–**19**), which exhibit potent activity in RNA replication assays, with the carboxamide group in particular imparting high potency but also high cytotoxicity [72].

**Figure 9:**
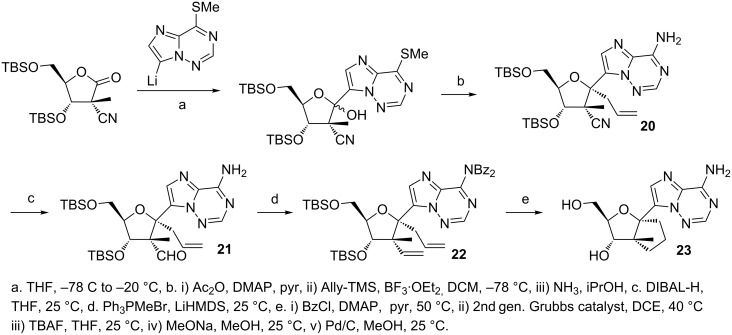
Synthesis of 1',2'-cyclopentyl C-nucleoside [73]. Functional groups at C1' and C2' were installed and employed for ring cyclization.

A further modification to the imidazo[2,1-*f*][1,2,4]triazine C-nucleoside scaffold was reported by Dang et al., wherein they synthesized a series of 2'-β-Me analogues possessing a 1',2' cyclopentyl ring (Figure 9) [73]. A representative synthesis (compound **23**) is shown in Figure 9, which involves installing an allyl group at C1' (**20**) and converting the C2'–CN to an aldehyde (**21**) followed by a Wittig reaction to install a second allyl group at C2' (**22**). Second generation Grubb’s catalyst was used for the ring formation, followed by hydrogenation to give the desired cyclopentane ring (**23**) [73]. The biological data of these compounds has yet to be reported.

**Figure 10:**
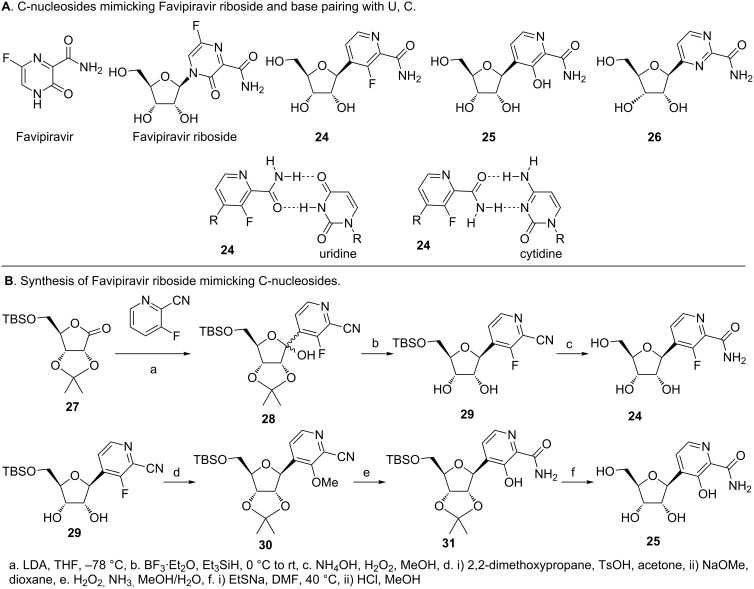
Anti-influenza C-nucleosides mimicking favipiravir riboside [74]. A. Structure of favipiravir and its riboside, which exhibits anti-influenza activity. C-nucleoside variants of favipiravir riboside and their base pairing with uridine and cytosine. B. Synthesis of C-nucleoside variants of favirpiravir starting from D-ribonolactone.

Wang et al. synthesized a series of pyridine and pyrimidine C-nucleosides (**24**–**26)** that mimic the riboside of favipiravir in their effort to develop novel anti-influenza compounds (Figure 10A) [74]. Protected D-ribonolactone **27** was treated with lithiated pyridine to obtain lactol **28** (Figure 10B). Deoxygenation and reduction gave **29**, wherein the isopropylidene group was also removed. Conversion of the cyano to an amide group, followed by removal of the silyl protecting group gave **24**, which proved to be the most promising compound. The fluorine on **29** was replaced with a methoxy group after re-installing the isopropylidene protecting group. The cyano group was then converted to an amide and the methoxy converted to a hydroxy group. Removal of the protecting groups on the sugar gave **25**, which exhibited potent activity against the H1N1 influenza strain (A/WSN/33) in cell based assays [74]. The pyrimidine compound **26** was synthesized using an identical approach and is not shown here. The activity of **24** and **25** as nucleosides was comparable to favipiravir and its riboside. Furthermore, they found that the triphosphate of **24** (**24**-TP) was incorporated opposite U and C of an RNA template by the influenza polymerase [74]. These experiments indicate that the H-bonding motifs of **24** allow it to mimic both A and G (Figure 10A) [74]. Despite the mis-incorporation, an unmodified sugar moiety may not result in obligate chain termination. While **24**-TP is incorporated opposite U and forms more of the full length product than terminated product, its incorporation opposite C results in greater truncated product. Thus, the putative mechanism of action of **24** is through mutagenesis of viral genomic RNA and inhibition of viral polymerase [74].

### Synthesis of C2'-substituted furanolactone

**Figure 11:**
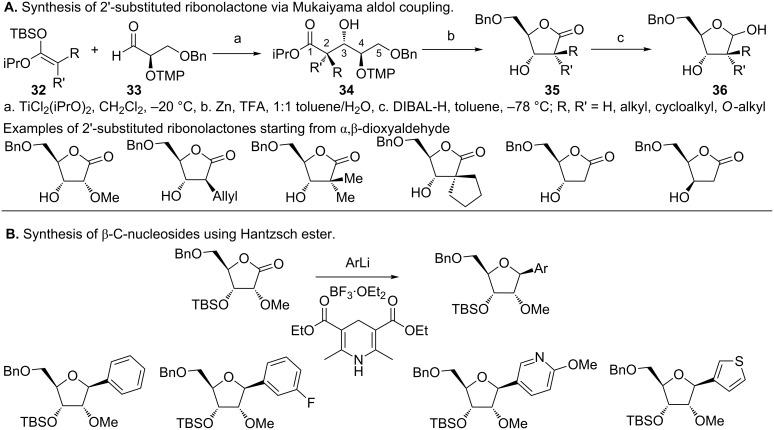
Alternative method for synthesis of 2'-substituted C-nucleosides [75]. A. Synthesis of C2'-substituted D-ribonolactone via Mukaiyama aldol reaction. A series of 2'-O-alkyl, alkyl, cycloalkyl and deoxy D-ribonolactone were synthesized using this method. B. Use of Hantzsch ester to obtain the β-anomer of C-nucleosides.

In view of sugar scaffolds possessing C2' substitutions and their value to drug design, a report by Peifer et al. on the synthesis of C2'-substituted ribonolactones is notable (Figure 11A) [75]. Their finding appends known methods of C2' substitution that involve conversion of the C2'-OH to a ketone followed by Me or F substitution [87–94]. Using the Mukaiyama aldol reaction, Peifer obtained a C2'-substituted ribonolactone, which can then be employed in C-nucleoside synthesis [75]. This involves condensation of alkyl-substituted silyl ketene acetals (**32**) with enantioenriched α-2,2,6,6-tetramethylpiperidinyl-β-benzyloxypropionaldehyde (**33**) in presence of TiCl_2_(OiPr)_2_ to give the β-hydroxyester **34** that is diastereomerically enriched [75,95]. Reductive cleavage of the 2,2,6,6-tetramethylpiperidinyl (TMP) group by Zn and trifloroacetic acid results in cyclization and formation of the C2'- substituted ribonolactone (**35**). TiCl_2_(OiPr)_2_ has been identified as the optimal Lewis acid for the synthesis of most ribonolactones with the exception of unsubstituted silyl ketene acetals (R = R' = H) that leads to stereochemical inversion at C3' [75]. The desired stereoselectivity for 2'-deoxy analogues was obtained when BF_3_·OEt_2_ was used. Furthermore, another route to the synthesis of C-nucleosides was demonstrated by direct addition of aryl lithium reagents to the 2'-OMe ribonolactone (Figure 11B). While the expected lactol was formed, deoxygenation by BF_3_·OEt_2_ and reduction in presence of the Hantzsch ester afforded the desired β-anomer, while the use of Et_3_SiH gave the α-anomer [75].

### Carbocyclic C-nucleosides

**Figure 12:**
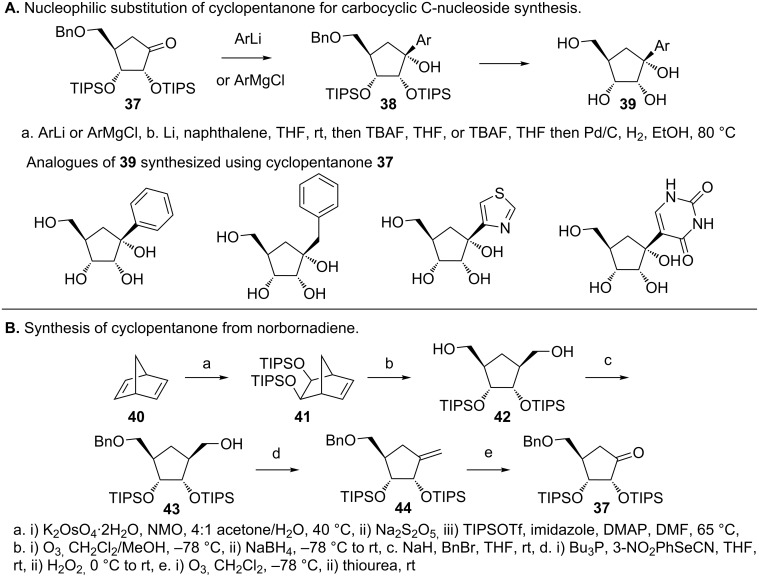
Synthesis of carbocyclic C-nucleosides using cyclopentanone [53]. A. Nucleophlic substitution on cyclopentanone gives C1'–OH carbocyclic C-nucleosides. B. Synthesis of cyclopentanone from norbornadiene and substituents that facilitate carbocyclic C-nucleoside syntheses.

In an attempt to synthesize carbocyclic C-nucleosides, Maier et al. found that reaction of aryl lithiums with pentanone **37** results in carbocyclic C-nucleosides with a C1'-hydroxy group (**38** and **39**, respectively, Figure 12A) [52–53]. They synthesized cyclopentanone **37** in 7 steps starting from norbornadiene (**40**, Figure 12B). Furthermore, silyl protection (TIPS) of the C2' and C3' was observed to be critical for the stability of **37** and to facilitate functional group interconversions as shown in Figure 12A [53].

**Figure 13:**
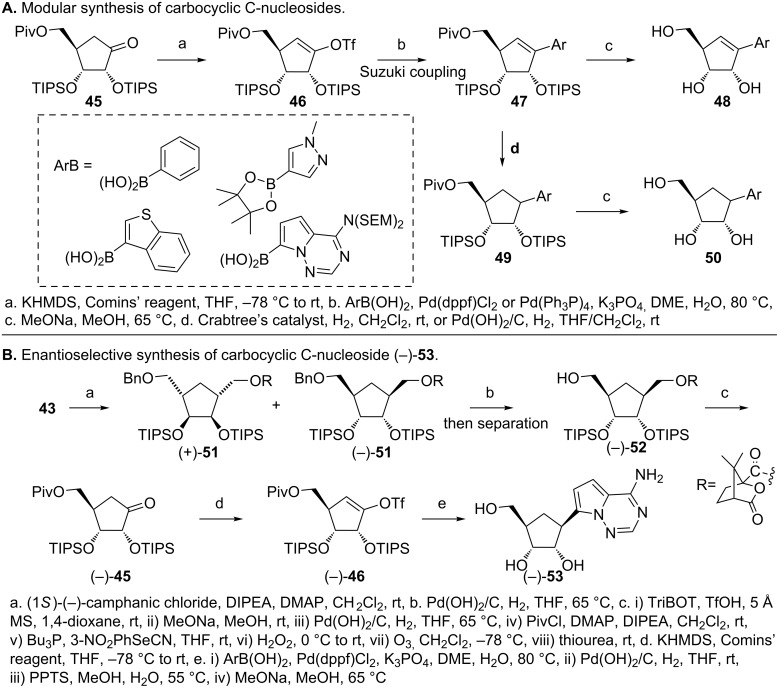
Synthesis of carbocyclic C-nucleosides via Suzuki coupling [53]. A. Synthesis of OTf-cyclopentene that enable Suzuki coupling and modular synthesis of carbocyclic C-nucleosides. B. Synthesis of enantiomerically pure 4-aza-7,9-dideazaadenine carbocyclic C-nucleoside.

In order to obtain carbocyclic C-nucleosides that resemble canonical nucleosides, Maier and coworkers synthesized a stable enol triflate (**46,** Figure 13A), which then enables Suzuki coupling and a modular synthesis of carbocyclic C-nucleosides [53]. The boronic acids/boronates (inset, Figure 13) of several (hetero)aryls were conducive to Suzuki coupling with the best result obtained when the C2', C3' and C5'–OHs were protected with TIPS and pivaloyl groups, respectively [53]. The cross-coupling reaction gave the unsaturated compounds (**47** and **48**), which, upon hydrogenation in presence of Crabtree’s catalyst, gave the saturated compounds with the desired diasteroselectivity (**49** and **50**). In the case of nitrogen containing heterocycles, Pd(OH)_2_ was found to be a suitable catalyst that gave a separable mixture of diastereomers (2:1). In addition, optically pure cyclopentanone (−)-**45** was obtained by converting the cyclopentane **43** to camphanates (**51,** Figure 13B) followed by separation of the diastereomers [53]. Subsequent synthesis of the enol triflate (−)-**46** (Figure 13B), Suzuki coupling and hydrogenation afforded the optically pure carbocyclic tubercidine analogue (−)-**53**. This compound has shown potent activity against breast cancer cell lines and human foreskin fibroblasts [53].

## Conclusion

With increasing reports of emerging and reemerging infectious diseases globally, there is a need to develop more effective and safer drugs. In that regard, C-nucleosides have recently shown great potential, which in turn, has resurrected interest in this class of molecules [29]. Several antiviral C-nucleosides have been discovered in the past five years and are now in advanced stages of clinical applications. The overarching features of these compounds with regards to changes in the nucleobase and sugars allow optimal interactions with enzymes resulting in potent and often times, selective, inhibitory activities [18,65,74,96]. As continuing efforts to design greater diversity in C-nucleosides, methods of their synthesis have become critical to more effective drug discovery. For example, the pyrrolo[2,1-*f*][1,2,4]triazine scaffold has been key to the discovery of several highly active molecules [53,69,71–73,86]. Modular and convergent synthetic routes have proved valuable in this regard both in terms of increasing diversity and reducing the time and length of the syntheses [70–73,76]. Efforts have been aided by advances in the synthesis of modified sugars and sugar mimics, particularly D-ribonolactone analogues [53,73,75,97]. Furthermore, chemical and theoretical studies have elucidated the mechanism and stereochemical preferences of reactions involving D-ribonolactone [80–81,84–85]. Therefore, the chemist has better control over the reactions with more predictable outcomes. In the coming years, new applications may be reported. Moreover, with the biological potential of C-nucleosides now being revisited, studies of naturally occurring C-nucleosides and their biosynthetic pathways have garnered renewed interest, as has the pursuit of new biosynthetic C-nucleosides [98–104]. Previously reported C-nucleosides are also being revisited and may be repurposed with increased knowledge of new biological targets [29,65,86,96]. In summary, these efforts, in concert with improved synthetic advances, provide strong impetus for the next wave of C-nucleoside design and the discovery of nucleoside therapeutics.
